# Safety and efficacy of low-dose intravenous arsenic trioxide in systemic lupus erythematosus: an open-label phase IIa trial (Lupsenic)

**DOI:** 10.1186/s13075-021-02454-6

**Published:** 2021-03-03

**Authors:** Mohamed Hamidou, Antoine Néel, Joel Poupon, Zahir Amoura, Mikael Ebbo, Jean Sibilia, Jean-Francois Viallard, Benjamin Gaborit, Christelle Volteau, Jean Benoit Hardouin, Eric Hachulla, François Rieger

**Affiliations:** 1 Department of Internal Medicine, CHU Nantes, Nantes Université, Nantes, France; 2 Department of Biological Toxicology, AP-HP, Lariboisière Hospital, University Paris VII, Paris, France; 3 Department of Internal Medicine 2, Centre National de Référence pour le Lupus, Institut E3M, Hôpital Pitié-Salpétrière, Paris, France; 4 Service de Médecine Interne, Aix Marseille Univ, APHM, CNRS, INSERM, CIML, Hôpital de la Timone, Marseille, France; 5 Department of Rheumatology, University of Strasbourg, Strasbourg, France; 6 Department of Internal Medicine, Haut-Lévêque University Hospital, Bordeaux, France; 7 Plateforme de Méthodologie et Biostatistiques, CHU Nantes, Université de Nantes, Nantes, France; 8 INSERM UMR 1246-SPHERE, Université de Nantes, Nantes, France; 9 Department of Internal Medicine, Centre de Référence des Maladies Autoimmunes Systémiques Rares du Nord et Nord-Ouest de France (CeRAINO), University of Lille, Lille, France; 10 MEDSENIC, SAS, a company with CNRS participation, Strasbourg, France

**Keywords:** Systemic lupus erythematosus, Autoimmune diseases, Treatment, Arsenic trioxide, Phase II clinical trial

## Abstract

**Background:**

Lupus animal model has shown that arsenic trioxide (ATO), a treatment of acute promyelocytic leukaemia, could be effective in SLE. This is the first clinical study to determine the safety and efficacy of a short course of intravenous ATO in patients with active SLE.

**Methods:**

This phase IIa, open-label, dose-escalating study enrolled 11 adult SLE patients with a non-organ threatening disease, clinically active despite conventional therapy. Patients received 10 IV infusions of ATO within 24 days. The first group received 0.10 mg/kg per injection, with dose-escalating to 0.15 mg/kg in a second group, and to 0.20 mg/kg in a third group. The primary endpoint was the occurrence of adverse events (AEs) and secondary endpoints were the number of SLE Responder Index 4 (SRI-4) responders at week 24 and reduction of corticosteroid dosage. In an exploratory analysis, we collected long-term data for safety and attainment of lupus low disease activity state (LLDAS).

**Results:**

Four serious AEs occurred (grade 3 neutropenia, osteitis, neuropathy), 2 of which were attributable to ATO (neutropenia in the 2 patients treated with mycophenolate). Two patients suffered a severe flare during the last 4 weeks of the trial. At W24, five patients among 10 were SRI-4 responders. Overall, mean corticosteroid dosage decreased from 11.25 mg/day at baseline to 6 mg/day at W24 (*P* < 0.01). In the long term, 6 patients attained LLDAS at W52, which continued at last follow-up (median LLDAS duration 3 years, range 2–4).

**Conclusions:**

A short course of ATO has an acceptable safety profile in SLE patients and encouraging efficacy.

**Trial registration:**

ClinicalTrials.gov, NCT01738360 registered 30 November 2012

**Supplementary Information:**

The online version contains supplementary material available at 10.1186/s13075-021-02454-6.

## Background

Systemic lupus erythematosus (SLE) is a heterogeneous autoimmune disease characterised by overproduction of type I interferons, hyperactivity of B cells with production of antibodies, presence of autoreactive T cells and activation of NETosis [1]. In SLE patients with persistent disease activity despite antimalarials and low dose steroids, quality of life is adversely affected by the unpredictable risk of flares and by drug toxicity and accumulation of organ damage. Belimumab is the only treatment approved since 2011 in refractory mild to moderate SLE, especially with high activity and positivity of biomarkers [2]. Currently, many controlled phase II/III trials evaluating targeted therapies have failed to meet their primary end point [3]. Drugs primarily used in onco-haematology may exert targeted or pleiotropic immunological effects and thus be effective in SLE. Unfortunately, phase III clinical trials evaluating rituximab failed to meet their primary end point [4, 5]. More recently, bortezomib, a proteasome inhibitor approved in multiple myeloma, demonstrated unacceptable toxicity in 12 SLE patients [6]. Trials evaluating new drugs such as Janus kinase or Bruton’s tyrosine kinase inhibitors are ongoing.

Arsenic trioxide (ATO), As_2_O_3_ has been approved for the treatment of acute promyelocytic leukaemia (APL) [7, 8], and more than 100 trials involving ATO are ongoing for treatment of solid and haematological neoplasias (myeloma, B cell neoplasia, etc.), in adults, as well as in elderly patients and children [9–11]. In recent years, ATO has been studied in several animal models of SLE [12, 13], systemic scleroderma [14] and chronic graft-versus-host disease (cGVHD) with scleroderma-like features [15]. Bobé et al. [12] demonstrated striking results with intraperitoneal injection of ATO treatment in the murine lupus model MRL-*lpr/lpr*, characterised by an autoimmune lymphoproliferative syndrome, with tissue infiltration by double-negative T lymphocytes, and lupus-like manifestations. As2O3 induced a rapid and marked decrease in the size of spleen and lymph nodes and suppressed skin lesions compared to PBS treated control MRL/lpr mice. It also inhibited auto-antibody production and immune complex deposition in the kidney and prevented nephritis. Furthermore, arsenic drastically improved the survival of treated mice. In this model, ATO did not act as a cytotoxic immunosuppressant. Rather, it exerted a selective pro-apoptotic effect of deletion of autoreactive lymphocytes, inhibited the production of autoantibodies, and reduced the levels of cytokines. In peripheral blood mononuclear cells of lupus patients, ATO reduces the expression level of IFN-gamma [16].

Thus, given the promising results in preclinical studies, we conducted this pilot open-label, dose-escalating multicentre phase IIa trial, to evaluate the safety and efficacy of a short course of ATO in the treatment of active SLE with inadequate responses to standard therapy.

## Methods

### Study design and patients

LUPSENIC was a phase IIa, open-label controlled, multicentre trial, evaluating dose-escalation intravenous ATO in SLE (EudraCT: 2012-002259-40; NCT: NCT01738360). The study was conducted between July 2013 and October 2015 and included patients from 6 university hospitals in France (Nantes, Lille, Paris, Bordeaux, Marseille, Strasbourg). This pilot trial was designed to examine the safety profile and tolerability of ATO as a primary objective, with efficacy as a secondary objective, in patients with active SLE.

All patients fulfilled at least four of the 11 American College of Rheumatology (ACR) criteria for SLE [17, 18], were aged 18 years or more and had a clinically active disease, defined by a Safety of Estrogens in Lupus Erythematosus: National Assessment–SLE Disease Activity Index (SELENA-SLEDAI) score > 6 [19], despite standard-of-care treatment. All patients were seropositive for anti-nuclear antibody (ANA) (≥ 1/80 in immunofluorescence assay). Patients with severe active renal or CNS disease, a history of cardiac disease, cytopenia with haemoglobin < 11 g/dl, neutrophil count < 1200/m^3^, and/or platelet count < 100,000/ml were excluded. Patients had to be receiving prednisone dose ≥ 10 mg/day and be refractory, for at least 1 year, or intolerant, to hydroxychloroquine (HCQ). Concomitant therapy with HCQ and/or immunosuppressive drugs (methotrexate [MTX], azathioprine [AZA], mycophenolate mofetil [MMF]) were allowed at stable doses for a minimum of 6 months before the start of study treatment Corticosteroid dosage tapering was left to investigators’ discretion, based on their clinical judgement.

The therapeutic scheme was designed on the basis of pharmacokinetics data obtained from APL treatments in previous studies: [20] patients were administered a loading dose of ATO on each of the first 4 days (as in-patients), then twice a week (as outpatients) during weeks 2 to 4, i.e. 10 IV infusions (duration: 4 h) within 24 days. To avoid the risk of QT prolongation, patients had to have normal kaliema and magnesemia at the start of, and during, the treatment period. Dose escalation was conducted using the continual reassessment method [21, 22]. The ATO doses were studied sequentially, patients receiving 0.1 mg/kg per injection, 0.15 mg/kg per injection or 0.20 mg/kg per injection (see online supplementary S1 and S2).

All patients provided written informed consent in accordance with the declaration of Helsinki. The study was approved by regional Human Subjects Research Protection Committee (*Comité de Protection des Personnes Ouest-I*, Tours hospital). All the safety data were submitted to an independent Data and Safety Monitoring Board (DSMB).

### Assessments

The primary endpoint was the occurrence of adverse events (AEs) and the secondary endpoints evaluated the efficacy of ATO. During the 24 weeks of the trial, patients made regular scheduled visits, at baseline and at weeks 8, 12, 16, 20, and 25. The last patient visit occurred in October 2015. Long-term follow-up data were updated in February 2018, in terms of safety (severe AE [SAE], death) and efficacy (lupus low disease activity state [LLDAS], severe flare, corticosteroid dosage).

### Primary and secondary outcome measures

The primary outcome measures for safety were the recording of AEs evaluated by NCI Common Terminology Criteria, including incidence, nature and severity of AEs, clinical laboratory abnormalities, vital signs, ECGs and physical examination findings. Serum creatinine, potassium and magnesium, complete blood cell count and transaminases were checked before each infusion of ATO and at each visit. The main secondary outcome was the efficacy of ATO on disease activity. Disease activity was evaluated using the SELENA-SLEDAI [19], the British Isles Lupus Assessment Group (BILAG) index [23], the physician’s global assessment (PGA) and clinical laboratory assessments, including proteinuria and serum biomarkers (anti-dsDNA antibodies, C3 and C4 complement, total IgG, IgA IgM dosage). Testing for anti-dsDNA antibodies was performed by Elisa or Farr test, according to local standard practice, using a single method for each patient.

Flares were defined according to the SELENA-SLEDAI Flare Index (SFI): [24, 25] flares were mild or moderate if changes in the SELENA-SLEDAI score were > 3 points or severe if the SELENA-SLEDAI score increased by 12 points. SELENA-SLEDAI, PGA and BILAG index scores and corticosteroid dosage were recorded every 4 weeks, as were AEs, vital signs, concomitant medications and laboratory results.

We assessed the SRI-4 response at week 24. SRI-4 responders were defined as having a reduction of at least 4 points in the SELENA-SLEDAI score, no new BILAG A or no more than one new BILAG B and no worsening in their PGA score [26]. Health-related quality of life was assessed with the 36-item Short-Form Health Survey (SF-36). Corticosteroid sparing was defined as a reduction in oral corticosteroid (OCS) dosage to 7.5 mg/day or less.

Arsenic level was determined in serum and erythrocytes by inductively coupled plasma-mass spectrometry before and after each IV infusion.

### Long term follow-up

In a long term follow-up analysis, we evaluated the number of patients who attained LLDAS within 52 weeks after ATO administration. LLDAS was defined as follows: SELENA-SLEDAI score ≤ 4 without major organ activity, no new disease activity, no new features of lupus disease activity compared with the previous assessment, PGA ≤ 1 on a 3-point scale, current prednisolone-equivalent dosage < 7.5 mg/d and well-tolerated standard maintenance dosages of immunosuppressive drugs and approved biologics [27]. We also evaluated time to first flare (according to SFI and BILAG) beyond W24 for SRI responders and patients who attained LLDAS at W52.

### Statistical analysis

Descriptive statistics are presented as median and interquartile range (IQR) for quantitative variables, or counts and percentages for categorical variables, unless otherwise indicated. No formal statistical calculation was performed to determine the necessary sample size. The continual reassessment method was performed with R v.3.1.1. The statistical significance of changes in SLEDAI, daily OCS dose and PGA compared to baseline values was tested using mixed model for repeated measurements. Analyses were performed with SAS v.9.

## Results

### Study population

Eleven patients were included in the study. All were Caucasians, eight were women and the mean age was 44 years. At entry in the study, all patients had active SLE in the mucocutaneous and/or musculoskeletal domain, were treated with OCS at doses ≥ 10 mg/day and antimalarials (except 1 patient with a history of APS-related maculopathy). Six among 11 had a concomitant immunosuppressive treatment (3 MTX, 2 MMF and 1 AZA). All patients had ANA ≥ 1/80, five had low C3 complement levels, three had low C4 levels and six had anti-dsDNA antibodies.

Four patients received 0.10 mg/kg per injection of ATO, four patients had 0.15 mg/kg per injection and three patients had 0.20 mg/kg per injection. Among the 11 included patients, one patient was excluded at day 17, from the study and from the dose escalation calculation with DSMB approval, because of protocol violation (administration of ATO despite grade 3 neutropenia).

### Safety profile

All the patients were evaluated for safety analysis. No dose-limiting toxicity (DLT) occurred and the study was discontinued when we reached 11 ATO-treated patients. Among the 11 included patients, two patients discontinued ATO after seven injections because of treatment-related SAEs. A description of AEs by System Organ Class is given in Table 1. Eighteen AEs were considered by the treating physicians to be related to the study drug and were generally mild to moderate. One patient had moderate hypomagnesemia and hypokaliema, five patients had diarrhoea and two patients had asymptomatic moderate QT prolongation (< 470 msec). Four SAEs occurred in four patients during the study, and two were formally considered by the treating physicians to be ATO-related. The latter occurred in two patients treated with ATO 0.15 and 0.20 mg/kg, respectively, and also receiving MMF, who experienced asymptomatic transient grade 3 neutropenia (neutrophil nadir at 0.69 and 0.51 G/L) leading to permanent discontinuation of ATO. Both patients showed complete spontaneous recovery in less than 5 days, without infection. There was no clear correlation between the arsenic levels reached and the observed toxicity. Another patient (ATO 0.2 mg/kg), had a grade 2 reversible sensory peripheral neuropathy, concomitant with acute hepatitis A virus infection. One patient (ATO 0.15 mg/kg) treated with a non-steroidal anti-inflammatory drug and 10 mg/day of prednisone had chronic cutaneous ulceration of a toe (Jaccoud arthropathy), which was complicated by an osteitis, requiring limited amputation of the distal phalanx.

**Table 1:** Arsenic trioxide safety at week 24

***Adverse event type***	*n*
***Total number of ATO-related AEs***	**22**
***Treatment-emergent SAEs***	**4**
Neutropenia*	2
Axonal neuropathy†	1
Osteitis‡	1
***AEs leading to ATO discontinuation***	
Neutropenia grade 3	2
***Treatment-related AEs (mild to moderate)***	**18**
Anaemia (grade 2)	2
Diarrhoea (grade 1)	5
Nausea	2
Hypomagnesaemia	1
Mild QT prolongation (grade 1)	2
Injection-related reaction	1
Injection site reaction	1
Hypotension	1
Flushing	1
Increased transaminases (grade 1)	1
Fatigue	1

* Neutropenia in 2 patients treated respectively with 0.15 and 0.20 mg/kg of ATO associated with MMF

^†^ Neuropathy associated with acute hepatitis A virus infection

^‡^ Osteitis of a toe in a patient with Jaccoud arthropathy

*AEs* adverse events, *SAE* severe adverse event, *ATO* arsenic trioxide, *MMF* mycophenolate mofetil

### Efficacy

#### Clinical efficacy

As mentioned previously, one patient was excluded due to protocol violation. Among 10 patients assessed for efficacy, nine patients received 10 ATO IV injections as planned in the protocol, and one patient discontinued ATO after seven injections, because of neutropenia related to ATO.

**Fig. 1:**
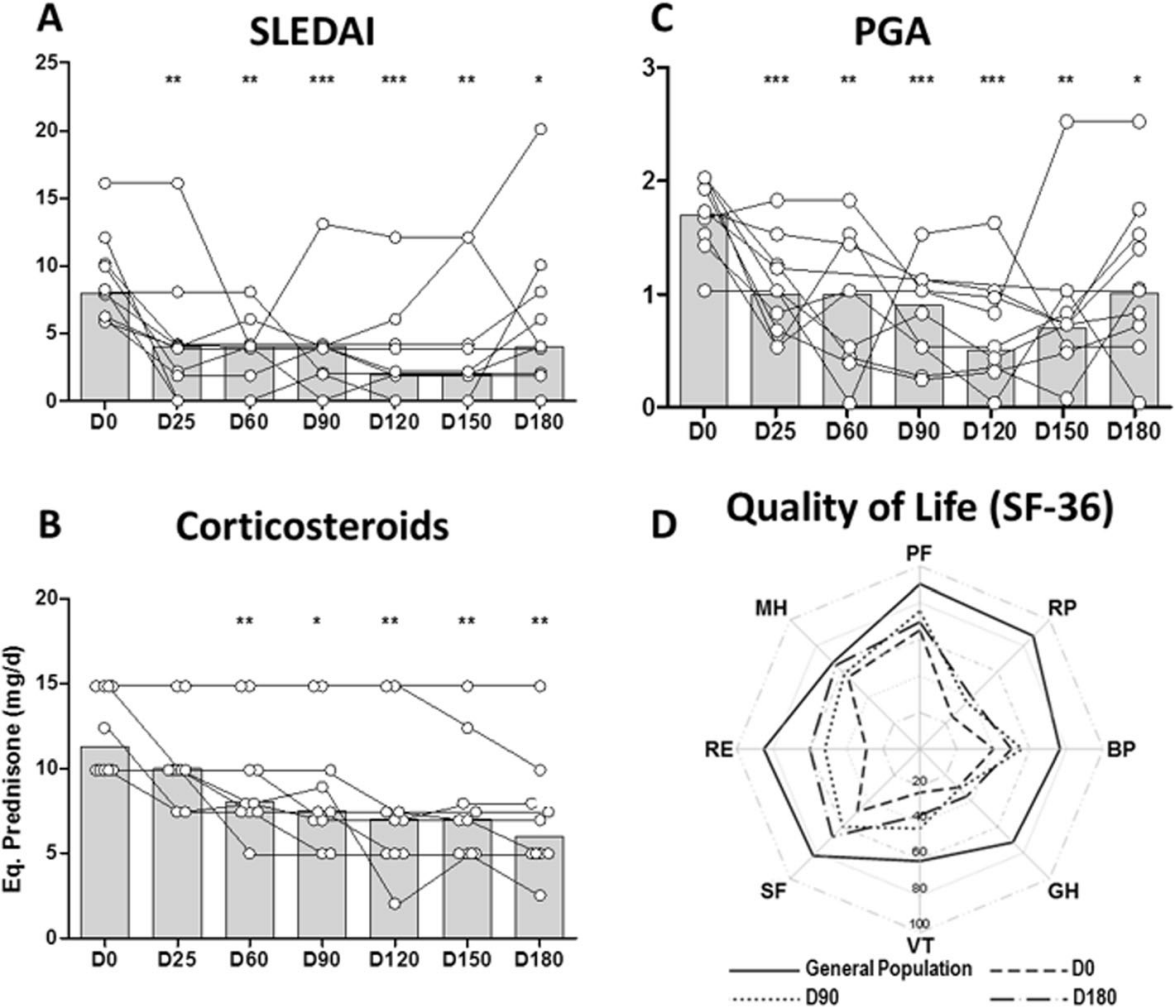
Clinical efficacy outcomes of ATO. Efficacy results after ATO infusion. Treatment was given over 21 days and changes in **a** SLEDAI score, **b** corticosteroid dose and **c** PGA score were recorded every 4 weeks over 24 weeks thereafter. Histograms depict the median. *P < 0.05, **P < 0.01, ***P < 0.001 (mixed model for repeated measurements). **d** Median Health-Related Quality of Life (HRQoL) scores for the 8 dimensions of the SF36 for the patients at D0, D90 and D180 and the score of a gender- and age-matched sample of individuals drawn from the general French population. We noted a marked increase between D0 and D90/D180 for all the dimensions of the HRQoL and globally stable scores between D90 and D180, which remained inferior to those of the general population. ATO, arsenic trioxide; Eq., equivalent; PGA, Physician Global Assessment; SLEDAI, Systemic Lupus Erythematosus Disease Activity Index

Five out of ten patients (50%) achieved a SRI-4 response at W24, three with 0.1 mg/kg ATO dosage and two with 0.20 mg/kg ATO dosage (Table 2). Median SLEDAI score at inclusion was 8 and decreased to 3.4 points at week 16 (Fig. 1a, *p* < 0.001). Two patients had a severe flare, one at W24 and the other W20, with BILAG A respectively in the renal and mucocutaneous domains, respectively. They received immunosuppressant after W24. Two other patients had BILAG B in the mucocutaneous domains. There were no significant differences in SRI-4 responder rates among the three ATO regimens.

**Table 2:** Patients baseline characteristics and ATO efficacy at week 24

			Baseline				ATO dosage mg/kg/d	Week 24			
Patient no., gender, age (years)	SLE duration (years)	Previous IS	Concomitant treatment	Symptoms	SLEDAI	OCS mg/d		Severe flare	SLEDAI	OCS mg/d	W24 SRI-4-R
1. F, 35	12	MTX	HCQ, THAL, MTX	A, C	**10**	**15**	**0.10**	**W24**	**8**	**10**	**No**
2. F, 41	23	MTX	HCQ	A, C	**12**	**15**	**0.10**	**No**	**2**	**2.5**	**Yes**
3. F, 49	29	anti-Fcg-R mAb	HCQ	A, C	**10**	**15**	**0.10**	**No**	**4**	**7**	**Yes**
4. M, 37	2	MTX	HCQ, MTX	A, C	**6**	**10**	**0.10**	**No**	**2**	**8**	**Yes**
5. F, 47	26	CYC, Ci, MMF, RTX, THAL	HCQ	A	**8**	**15**	**0.15**	**No**	**10**	**15**	**No**
6. F, 30	4	BELI, MTX, EPRA, LEFLU,	HCQ	A, C	**6**	**10**	**0.15**	**No**	**6**	**7.5**	**No**
7. F, 50	14	MTX	HCQ	A	**8**	**12.5**	**0.15**	**W20**	**20**	**2**	**No**
8. H, 53	3	MTX	HCQ, MTX	A, C	**8**	**10**	**0.20**	**No**	**0**	**5**	**Yes**
9. F, 45	15	MTX, AZA, CYC	MMF	A, C	**16**	**10**	**0.20**	**No**	**4**	**5**	**Yes**
10. F, 23	6	AZA	HCQ, AZA	A, C	**6**	**10**	**0.20**	**No**	**4**	**5**	**No**

*ATO* arsenic trioxide, *AZA* azathioprine, *anti-Fcg-R mAb* anti-Fc gamma-receptor monoclonal antibody, *BELI* belimumab, *Ci* ciclosporine, *CYC* cyclophosphamide, *EPRA* epratuzumab, *HCQ* hydroxychloroquine, *IS* immunosuppressant, *LEFLU* leflunomide, *MTX* methotrexate, *MMF* mycophenolate mofetil, *OCS* oral corticosteroids, *RTX* rituximab, *THAL* thalidomide, *A* articular, *C* cutaneous, *SLEDAI* Systemic Lupus Erythematosus Disease Activity Index, *SRI-4-R* SLE response index-4 response

Tapering of OCS was done at the treating physician’s discretion. The median dosage of prednisone decreased from 12.25 mg/day at baseline to 6 mg/day at W24 (Fig. 1b, *p* = 0.0046). At W24, seven out of 10 patients reached the 7.5 mg/day target-dose.

#### Biomarkers and ATO pharmacokinetics

**Fig. 2:**
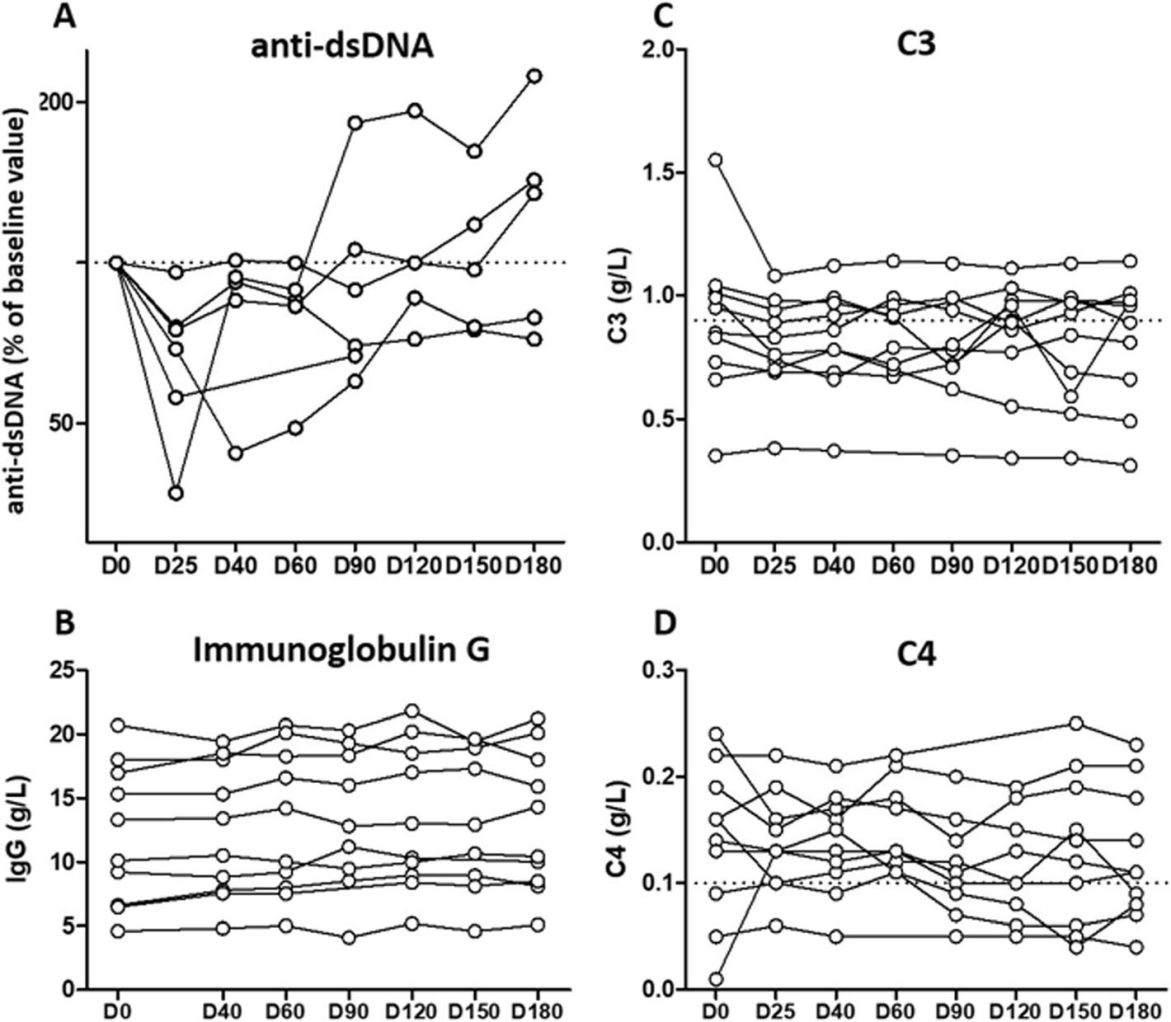
Immunological effects of ATO. Changes in **a** anti-ds-DNA, **b** immunoglobulin G, **c** complement factor 3 and **d** complement factor 4 levels over 24 weeks after ATO infusion. Each dot represents an individual measurement. Changes in anti-dsDNA level are represented as variation from baseline in 6 patients with detectable anti-dsDNA at baseline. Dotted lines represent the lower limit of normal complement level

**Fig. 3:**
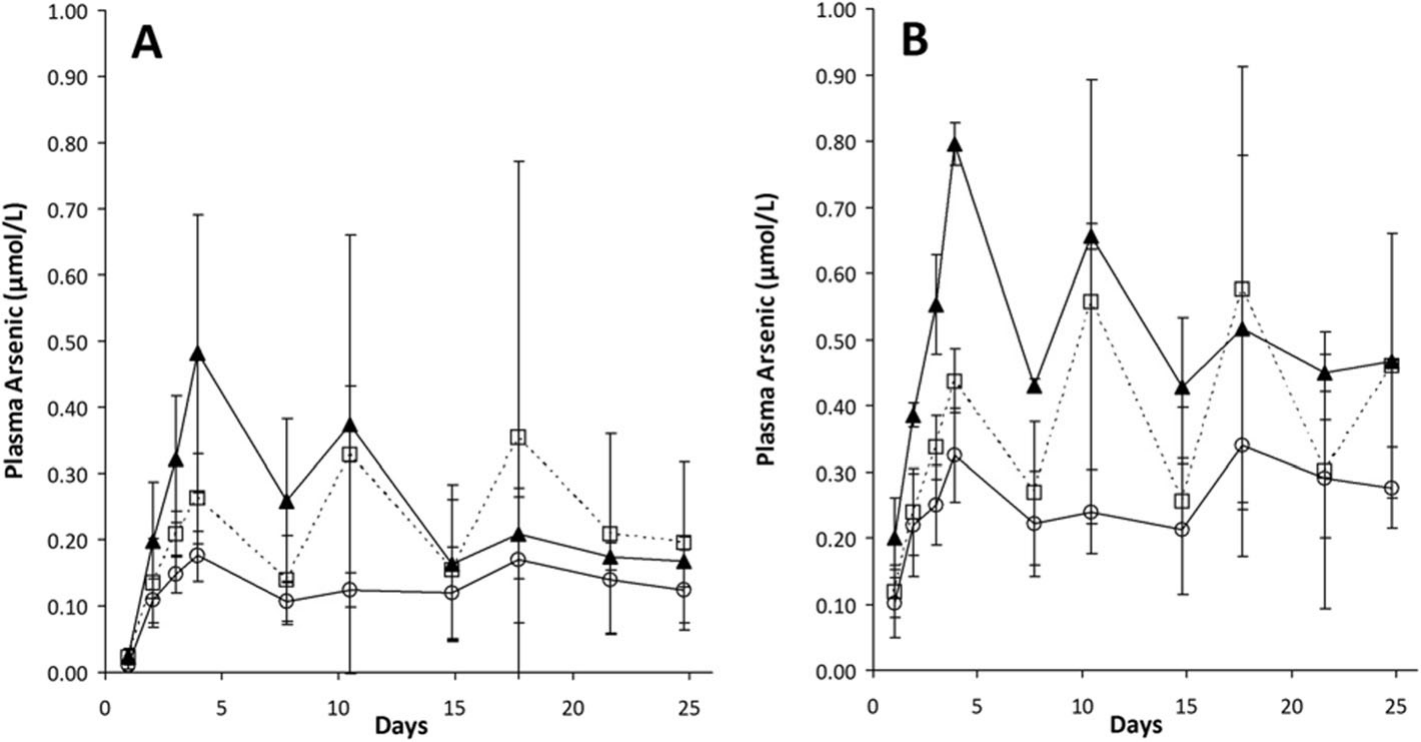
ATO pharmacokinetics. Plasma arsenic concentrations (mean +/− SD) in the three groups of ATO dosage before (**a**) or at the end of the perfusion (**b**). White circle indicates 0.10 mg/kg, white square indicates 0.15 mg/kg, and black triangle indicates 0.20 mg/kg

Among the six patients with detectable anti-dsDNA antibodies at baseline, a trend towards an initial decrease was observed from W4 to W8 (Fig. 2a). Levels of C3, C4, and IgG were not modified (Fig. 2b and c); levels of IgA and IgM were not modified (data not shown). Ro/SSA and anticardiolipin antibodies likewise remained unchanged (data not shown). Plasma arsenic concentrations were measured just before (residual) and at the end of the infusion for the three groups of ATO dosage (Fig. 3). During the induction phase (D1-D4), we observed a regular increase in arsenic concentrations in the three groups proportionally to the amount of ATO injected. At D8, arsenic concentrations decreased and stayed relatively stable between D8 and D25, with, large inter-individual variations after D8, especially in the 0.15 mg/kg group. Pharmacokinetic data did not show any correlation between plasma ATO levels and safety and efficacy (data not shown).

#### Long-term follow-up

Long-term follow-up data are reported in Table 3. One year after ATO administration, six out of 10 patients attained LLDAS with no further therapeutic intervention, and this state persisted at the last follow-up (median duration 45 months, range 34–51). At last follow-up, median OCS dosage of these patients was 2.8 mg/day (range 0–5). In terms of safety, no SAE related to ATO treatment was observed. A 34-year-old patient died from small-cell lung cancer 2 years after ATO administration. She was a tobacco smoker and had received thalidomide and immunosuppressive drugs for a long time, before and after ATO administration. ATO was deemed not to have been responsible by the Pharmacovigilance Team.

**Table 3:** Long-term follow-up

Patient no., gender, age (years)	ATO Dosage mg/kg/d	Week 24					Post-trial long-term follow-up					
		W24 treatment	SLEDAI score	OCS mg/day	W24 SRI-4-R	W24 LLDAS	W52 LLDAS	Severe Flare	At last follow-up			
									sustained LLDAS	OCS mg/day	LLDAS duration	Treatment
1. F, 35	**0.10**	HCQ, THAL, MTX	8	10	No	No	No	W24, C	No	–	–	–
2. F, 41	**0.10**	HCQ	2	**2.5**	**Yes**	**Yes**	**Yes**	**No**	**Yes**	0	**4 y 3 m**	HCQ
3. F, 49	**0.10**	HCQ	4	**7**	**Yes**	**Yes**	**Yes**	**No**	**Yes**	5	**4 y 2 m**	HCQ
4. M, 37	**0.10**	HCQ, MTX	2	**8**	**Yes**	**No**	**Yes**	**No**	**Yes**	0	**3 y 5 m**	HCQ, MTX
5. F, 47	**0.15**	HCQ	10	15	No	**No**	**Yes**	**No**	**Yes**	5	**4 y**	HCQ
6. F, 30	**0.15**	HCQ	6	**7.5**	No	No	**Yes**	**No**	**Yes**	7	**2 y 10 m**	HCQ
7. F, 50	**0.15**	HCQ	20	2	No	No	No	W20, R	No	–	–	–
8. H, 53	**0.20**	HCQ, MTX	0	**5**	**Yes**	**Yes**	**Yes**	**No**	**Yes**	0	**3 y 6 m**	HCQ, MTX
9. F, 45	**0.20**	MMF	4	**5**	**Yes**	**Yes**	No	W100, R	No	–	–	–
10. F, 23	**0.20**	HCQ, AZA	4	5	No	No	No	W80, R/H	No	–	–	–

*ATO* arsenic trioxide, *AZA* azathioprine, *HCQ* hydroxychloroquine, *MTX* methotrexate, *MMF* mycophenolate mofetil, *OCS* oral corticosteroids, *THAL* thalidomide C, cutaneous, *R* renal, *H* heart, *y* years, *m* months, Lupus Low Disease Activity State, *LLDAS* SLEDAI, Systemic Lupus Erythematosus Disease Activity Index, *SRI-4-R* SLE response index-4 response

## Discussion

There is a significant unmet need for new agents for treating refractory SLE [3]. In this pilot study, we report the first-in-human assessment of the effect of ATO in an autoimmune disease, namely SLE. Our first finding is that a short course of ATO at a low dose demonstrated an acceptable safety and tolerability profile in active SLE patients, with skin and joint manifestations, steroid-dependant and unresponsive to HCQ and immunosuppressive treatments.

In onco-haematology, ATO is administered at 0.15 mg/kg daily, for at least 5 weeks (i.e. 25 infusions), followed by maintenance therapy [27]. In our trial, patients only received 10 infusions, without maintenance, which resulted in a low total cumulated dose (1–2 mg/kg). Interestingly, we noted that these low cumulative doses of ATO resulted in an encouraging rate of response in patients with steroid-dependent active SLE despite standard-of-care treatment. Five (50%) patients had a 24-week SRI-4 response. Seven among 10 patients could reduce corticosteroid dose below 7.5 mg/day. An improvement of quality of life was also observed. We noted a decrease in clinical SLEDAI score at W12, suggesting a rapid effect of ATO in our chosen regimen and its potential use as an induction treatment. In contrast and despite a trend towards a short-term decrease of anti-dsDNA antibodies, ATO treatment did not significantly modify complement or Ig levels.

Unexpectedly, a long-term follow-up update revealed that, in spite of the short-duration treatment and steroid tapering, 6 out of 10 patients achieved LLDAS within 52 weeks after ATO, which persisted at the last follow-up (Table 3), up to 4 years after ATO administration. At last follow-up, 5 patients received ≤ 5 mg/day prednisone. LLDAS is a meaningful marker of response to treatment, correlated with a decrease in damage progression, and could be included in the assessment of future randomised controlled trials [28–31].

Four patients exhibited SAEs, only 2 of which were formally attributed to ATO, both corresponding to a grade 3 neutropenia, reversible with ATO withdrawal. Neutropenia, an expected AE of ATO and MMF, occurred in the two patients concomitantly treated with MMF. Thus, we call for caution in the event of an association of ATO with MMF. Most of the other AEs were mild and resolved after drug discontinuation. One 34-year-old patient developed small-cell lung cancer 2 years after discontinuation of ATO. She was a smoker, which remains the key risk factor for lung cancer in lupus patients [32]. In the onco-haematological setting, long-term follow-up of ATO-treated patients revealed no major chronic AEs, secondary carcinoma or arsenic retention [33].

During the daily ATO course (D0-D8), pharmacokinetic analysis was consistent with data obtained in APL [19]. DLT was not reached and no dose-related trends in safety and efficacy were noted between the three dosages. Thus, a dosage of 0.15 mg/kg seems adapted for further studies in SLE and other systemic auto-immune diseases.

Data regarding the immunological effects of ATO are contradictory*.* In the treatment of APL, ATO has a specific pro-apoptotic effect on leukaemic cells and decreases the amount and inhibits the function of Treg cells [34]. However, in a murine islet allotransplantation model, ATO inhibited immune rejection and prolonged islet allograft survival by increasing the proportion of Foxp3+ Treg cells, inhibiting proliferation of T lymphocytes and decreasing B lymphocytes [35]. ATO, when combined with co-stimulation blockade, prolongs the survival of cardiac allografts in alloantigen-primed mice. In this model, splenic CD4(+) or CD8(+) memory T cells were significantly reduced, while the expression of Tregs was enhanced [36]. Arsenic has also been shown to reduce dendritic cell cytokine production and the ability of dendritic cells to activate T cells in vitro [37]. Recently, Ye and coll. showed that As2O3 impaired activated pDCs of healthy donors to promote CD4 T cell proliferation and Th1/Th22 polarizations. At clinically relevant concentrations, As2O3 inhibits preferentially IFN-a secretion, via IRF7 and plasmablast differentiation of B cells. They also observed on PBMC of untreated systemic sclerosis patients, that similarly to healthy pDCs, As2O3 induced preferential inhibition of IFN-a secretion, proapoptotic effects and regulatory phenotype in SSc [38].

It is well established that dysregulation of oxidative stress is present in SLE [39]. In addition, ATO may be a potential immune modulator for treatment of rheumatoid arthritis, that helps to balance of Treg and Th17 cells through modulating STAT3 [40]. Interestingly, an altered Th17/Treg balance is also present in SLE, and could be a target of ATO, through IL-17 [41].

The scheme tested herein as IV induction therapy (daily IV infusion during 4 days, then infusions twice a week until week 4) was well accepted by patients. In the future, oral ATO, currently used in APL [42, 43], could be a good option to test in autoimmune diseases.

The major limitations of our study are the small sample size, the absence of a control group and the absence of in-depth immunomonitoring, such as the type 1 interferon signature.

## Conclusions

In this pilot clinical trial, a short course of intravenous ATO demonstrated an acceptable safety profile with an encouraging efficacy in SLE patients. These preliminary results support further evaluation in larger clinical trials to better define the benefit–risk of ATO in SLE and other systemic immune-mediated diseases.

## Supplementary Information

**Additional file 1: Figure S1.** LUPSENIC study scheme. A loading dose of ATO was administered intravenously each of the first 4 days (as in-patients), then twice a week (as outpatients) during weeks 2 to 4. **Figure S2.** Dose escalation scheme (Continual Reassessment Method): The ATO doses were studied sequentially; patients were planned to receive 0.1 mg/kg, 0.15 mg/kg or 0.20 mg/kg. If a patient experienced possible dose limiting toxicity (DLT), defined as grade 3 non reversible AE and/or grade 4 toxicity or death), a mathematical model was used to estimate the observed toxicity probability and determine the ATO dose for the next included patient. The maximum tolerated Dose (MTD) had been defined as the dose at which a DLT occurred in 10% of patients.

## Data Availability

The datasets analysed during the current study are available from the corresponding author on reasonable request.

## Abbreviations

ACR: American College of Rheumatology
ANA: Anti-nuclear antibodies
ATO: Arsenic trioxide
APL: Acute promyelocytic leukaemia
AZA: Azathioprine
BILAG: British Isles Lupus Assessment Group
cGVHD: Chronic graft versus host disease
DLT: Dose-limiting toxicity
HCQ: Hydroxychloroquine
IV: Intravenous
LLDAS: Lupus low disease activity state
MMF: Mycophenolate mofetil
MTX: Methotrexate
OCS: Oral corticosteroids
PGA: Physician’s global assessment
SELENA-SLEDAI: Safety of Estrogens in Lupus Erythematosus: National Assessment–SLE Disease Activity Index
SLE: Systemic lupus erythematosus
SFI: SELENA-SLEDAI Flare Index
SRI-4-R: SLE response index-4 response
