# Supplementary material for: Safety and efficacy of low-dose intravenous arsenic trioxide in systemic lupus erythematosus: an open-label phase IIa trial (Lupsenic)
Source: Arthritis Res Ther. 2021 Mar 3;23:70. doi: 10.1186/s13075-021-02454-6 (PMC7927234; doi:10.1186/s13075-021-02454-6)
Supplement: Supplementary file 1 — Additional file 1: Figure S1. LUPSENIC study scheme. A loading dose of ATO was administered intravenously each of the first 4 days (as in-patients), then twice a week (as outpatients) during weeks 2 to 4. Figure S2. Dose escalation scheme (Continual Reassessment Method): The ATO doses were studied sequentially; patients were planned to receive 0.1 mg/kg, 0.15 mg/kg or 0.20 mg/kg. If a patient experienced possible dose limiting toxicity (DLT), defined as grade 3 non reversible AE and/or grade 4 toxicity or death), a mathematical model was used to estimate the observed toxicity probability and determine the ATO dose for the next included patient. The maximum tolerated Dose (MTD) had been defined as the dose at which a DLT occurred in 10% of patients. [file 13075_2021_2454_MOESM1_ESM.docx]

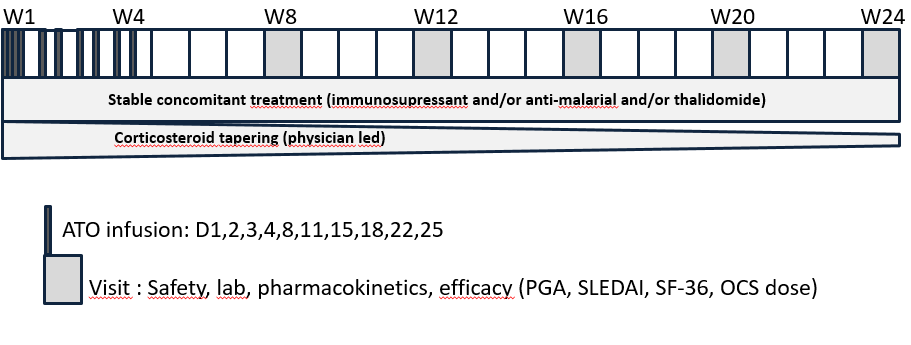


**Figure S1.** **LUPSENIC study scheme**. A loading dose of ATO was administered intravenously each

of the first 4 days (as in-patients), then twice a week (as outpatients) during weeks 2 to 4.


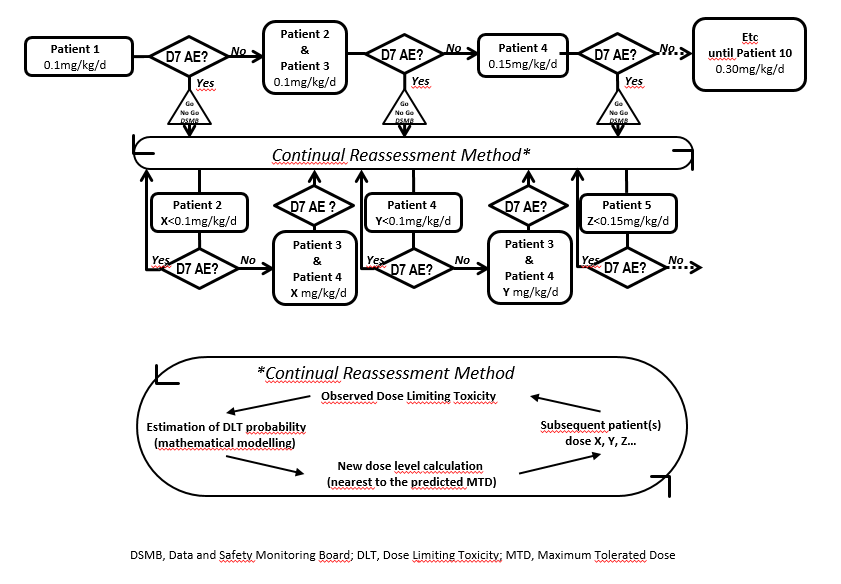


**Figure S2.** **Dose escalation scheme (Continual Reassessment Method):** The ATO doses were studied sequentially; patients were planned to receive 0.1 mg/kg, 0.15 mg/kg or 0.20 mg/kg. If a patient experienced possible dose limiting toxicity (DLT), defined as grade 3 non reversible AE and/or grade 4 toxicity or death), a mathematical model was used to estimate the observed toxicity probability and determine the ATO dose for the next included patient. The maximum tolerated Dose (MTD) had been defined as the dose at which a DLT occurred in 10% of patients.
